# Changes in bone mineral density and fractures during 2 years of low-dose glucocorticoid treatment for rheumatoid arthritis: a systematic literature review and individual participant data meta-analysis

**DOI:** 10.1136/rmdopen-2025-006615

**Published:** 2026-05-07

**Authors:** Andriko Palmowski, Tobias Haugegaard, Ingiäld Hafström, Henning Bliddal, Judith Oldenkott, Siegfried Wassenberg, Ernest Choy, John Kirwan, Robin Christensen, Maarten Boers, Frank Buttgereit

**Affiliations:** 1 Department of Rheumatology and Clinical Immunology, Charite - Universitatsmedizin Berlin, Berlin, Germany; 2 Section for Biostatistics and Evidence-Based Research, the Parker Institute, Bispebjerg and Frederiksberg Hospital, Frederiksberg, Denmark; 3 Epidemiology and Health Services Research, German Rheumatism Research Center (DRFZ), Institute of the Leibniz Association, Berlin, Germany; 4 Berlin Institute of Health (BIH) at Charité, Universitätsmedizin Berlin, Berlin, Germany; 5 Division of Gastroenterology and Rheumatology, Department of Medicine Huddinge, Karolinska Institutet, and Karolinska University Hospital, Stockholm, Sweden; 6 Rheumazentrum Ratingen, Ratingen, Germany; 7 Cardiff University, Cardiff, UK; 8 University of Bristol, Bristol, UK; 9 Research Unit of Rheumatology, Department of Clinical Research, University of Southern Denmark, Odense University Hospital, Odense, Denmark; 10 Department of Epidemiology and Data Science, Amsterdam UMC, Amsterdam, The Netherlands; 11 Translational Rheumatology, German Rheumatism Research Center (DRFZ), A Leibniz Institute, Berlin, Germany

**Keywords:** Epidemiology, Glucocorticoids, Arthritis, Rheumatoid, Osteoporosis

## Abstract

**Objective:**

To evaluate the effects of 2 years of low-dose glucocorticoid (GC) treatment on bone mineral density and fracture risk in patients with rheumatoid arthritis (RA).

**Methods:**

We performed a protocolised (dx.doi.org/10.17504/protocols.io.6qpvr3ombvmk/v1) systematic literature review and individual participant data meta-analysis of randomised trials in early and established RA, which compared GCs at ≤7.5 mg prednisone equivalent/day with placebo or standard of care. All patients could receive background treatment with disease-modifying antirheumatic drugs. Changes in lumbar spine and femoral bone density and participants with ≥1 clinical fracture over 2 years in intention-to-treat analyses were coprimary endpoints. Main analyses were based on one-stage models; I² was estimated from two-stage models. Missing data were handled using multiple imputation. Several sensitivity analyses assessed the robustness of our results.

**Results:**

Out of 2336 articles, five out of six identified trials provided individual participant data (1112 participants). Greater bone loss was observed at the lumbar spine in the GC compared with the control group (−0.021 g/cm²; 95% CI −0.037 to −0.005; p=0.034; I²=31%) but not at the femur (0.004 g/cm²; 95% CI −0.008 to 0.016; p=0.47; I²=0%). Subgroup analyses did not reveal groups particularly susceptible to GC-induced bone loss at the lumbar spine. 35 participants experienced ≥1 fracture; fracture risk was comparable in both groups. Sensitivity analyses yielded consistent results.

**Conclusion:**

Low-dose GCs, used for 2 years to treat RA, lead to bone loss at the lumbar spine but not at the femur.

**WHAT IS ALREADY KNOWN ON THIS TOPIC**

- Low-dose glucocorticoids (≤7.5 mg/day prednisone equivalent) are frequently used as a long-term cotreatment for rheumatoid arthritis in real-world settings.
- Trials and observational studies disagreed on the effect of low-dose glucocorticoids on bone health in patients with rheumatoid arthritis.

**WHAT THIS STUDY ADDS**

- In this systematic review and individual participant data meta-analysis, low-dose glucocorticoids, taken over 2 years to treat rheumatoid arthritis, had a small but statistically significant impact on the bone mineral density of the lumbar spine; femoral bone density was not affected.

**HOW THIS STUDY MIGHT AFFECT RESEARCH, PRACTICE OR POLICY**

- Our results serve to inform clinicians and patients about bone-related aspects of the benefit-risk ratio of low-dose glucocorticoids in patients with rheumatoid arthritis.
- Depending on each patient’s individual osteoporosis risk profile, the negative effect of low-dose glucocorticoids on the bone mineral density at the lumbar spine might or might not be clinically relevant.

## Background

 Glucocorticoids (GCs) such as prednisone are commonly used to treat rheumatoid arthritis (RA),^1^ but their benefit–risk ratio, especially if used at a low dose (ie, ≤7.5 mg/day prednisone equivalent^2^), is still controversially debated. While the American College of Rheumatology conditionally recommends against using GCs when initiating a disease-modifying anti-rheumatic drug (DMARD),^3^ the European Alliance of Associations for Rheumatology (EULAR) suggests that short-term GCs should be considered.^4^ A EULAR task force concluded that most patients requiring long-term doses of ≤5 mg/day have an acceptably low risk of harm.^5^ These diverging recommendations cause insecurity among practising rheumatologists worldwide.

Insufficient evidence regarding GC-related adverse events is the main reason for this controversy. Osteoporosis is a common comorbidity in RA and increases morbidity and mortality. It was among the most worrisome adverse events of GC treatment in a survey of rheumatologists and patients.^6^ Prior observational studies and randomised controlled trials (RCTs) disagreed on the effect of low-dose GCs on osteoporosis and fracture risk in patients with RA.^7^,^10^ Observational studies might overestimate the risk increase associated with GCs due to confounding,^11 12^ and RCTs were underpowered to reliably address safety concerns. Meta-analyses of trials can improve statistical power and overcome limitations of observational studies at the same time. In a prior study using RCT data to investigate the increase in blood pressure and body weight gain during low-dose GC treatment, we found that observational studies had overestimated both.^13^

We performed a systematic literature review and individual participant data (IPD) meta-analysis of RCTs to investigate the effect of low-dose GCs over 2 years on bone mineral density (BMD) and the risk of fractures in patients with RA. Additionally, by aggregating IPD, we planned to identify subgroups especially vulnerable to GC-induced bone loss in order to guide clinicians as to which patients may need closer monitoring or earlier treatment with specific anti-osteoporotic drugs.

## Methods

This study is being reported in accordance with Preferred Reporting Items for Systematic Reviews and Meta-Analyses-IPD reporting guidelines.^14^ A detailed protocol for this study was registered with protocols.io (dx.doi.org/10.17504/protocols.io.6qpvr3ombvmk/v1) before the study was initiated. Deviations from the protocol are outlined in online supplemental table S1.

### Data sources and search strings

MEDLINE (via PubMed), Embase (via Ovid) and the Cochrane Central Register of Controlled Trials (via Cochrane Library) were searched in accordance with current recommendations outlined in the Cochrane Handbook^6^ on 3 July 2023. Search strings can be found in online supplemental table S2. Additionally, references of included studies were screened.

### Eligibility

We included RCTs in RA that compared low-dose GC treatment (defined as 7.5 mg/day prednisone equivalent or less^2^) over at least 2 years with placebo or any other non-GC control treatment (eg, standard of care); there were no language restrictions. Concomitant treatment with DMARDs was allowed. Participants with both early and established RA were included. GC dose was converted to prednisone equivalent doses.^15^ Monthly treatment with depot intramuscular GCs was allowed and converted to an average daily dose.

### Study selection

Retrieved articles were imported into EndNote software (Clarivate Analytics, Philadelphia, Pennsylvania, USA). First, duplicates were eliminated. Subsequently, the articles were screened for inclusion or exclusion, first by title and abstract and then in full. The screening was performed independently by two reviewers (JO and AP). Finally, a consensus on study inclusion was reached, with discussions involving a third reviewer (FB) if necessary.

#### Data collection, items and management

First and/or last authors of all included studies were contacted to obtain IPD. The requested data were defined and outlined in our study protocol. For data extraction, harmonisation and management, we used Microsoft Excel (Microsoft, Redmond, Washington, USA) and SPSS (IBM, Version 28.0.1.0) software. Data integrity was checked by comparing the data received to published trial results.

### Outcome measures and endpoints

Changes in BMD (in g/cm^2^, as measured by dual X-ray absorptiometry) and participants with ≥1 fracture (clinical or symptomatic) during the 2-year study period from baseline until 2 years were defined as coprimary endpoints. Lumbar spine and femoral bone density were analysed separately. Femoral bone density included the bone density of the femoral neck and the total hip, as not all were available from all trials.

### Risk of bias within trials

We decided a priori in our protocol not to perform assessments of the risk of bias within trials because of the following reasons:

- The outcomes assessed in this study have not all been published in the main trial articles, making it difficult to assess certain domains of the Cochrane RoB 2 tool.^16^
- Principal investigators of included trials are coauthors of the present study, and a risk of bias assessment might itself be seen as biased.

#### Risk of bias across trials

We planned to assess publication bias in our coprimary outcomes by visual inspection of funnel plots, but the number of included studies was deemed too low for such an assessment.

### Statistical analysis

Trials that did not provide IPD were not included in quantitative synthesis. Baseline characteristics were summarised stratified by group (GC vs control) in numbers (and percentages), means (SD) or medians (and inner quartiles), depending on the variable’s distribution. As suggested during peer review, means (SD) are also reported for variables not following a normal distribution. I² was estimated from standard random-effects meta-analysis models (by performing a two-stage analysis^17^). All p values and 95% CIs were two-sided, the threshold for statistical significance α was set at 0.05.

Main analyses of the coprimary endpoints were based on one-stage models^17^ and the intention-to-treat approach, meaning that all patients were included in the statistical analyses regardless of any protocol violations or loss to follow-up. Missing outcome data were handled via multiple imputation by chained equations under a missing-at-random assumption, with a single imputation model applied consistently across all three coprimary analyses. All missing baseline values were replaced by the grand mean. The imputation model included study identifier, treatment allocation, fracture at follow-up and follow-up lumbar spine and femoral BMD, with each follow-up outcome used as an auxiliary variable for the others. Baseline bone measures included lumbar spine and femoral BMD at baseline. Baseline demographic, lifestyle and disease-related covariates included age, sex, body weight, body mass index, smoking status, disease duration, disease activity, pain severity, functional status and inflammatory and serological markers. Predictive mean matching was used for follow-up lumbar spine and femoral BMD, while a logistic model was used to impute fractures at follow-up. Changes in lumbar spine and femoral BMD were calculated within each imputed dataset, using the imputed follow-up scores.

R software (V.4.4.2) with packages mice, lme4 and emmeans was used for statistical analyses. For changes in bone density, an analysis of covariance (ANCOVA) model was employed. This model included treatment (two levels), baseline value of the outcome (one for each participant) and trial ID as terms. Trial ID was treated as a random effect, accounting for clustering of patients within trials. For fractures, a logistic regression model that included similar terms was employed. Least squares means were obtained from these models, and differences (with 95% CIs) were estimated at follow-up; ie, least squares means were estimated repeatedly, with missing outcome data imputed five times from patients receiving the same randomised treatment; results were combined using Rubin’s rules.

### Subgroup analyses

To identify subgroups especially vulnerable to bone loss at the lumbar spine, various baseline characteristics were assessed as effect modifiers: seropositivity (rheumatoid factor and/or anti-citrullinated protein antibody), smoking status, GC dose (5 mg/day vs 7.5 mg/day), baseline bone density, weight, disease activity (as measured by Disease Activity Score 28 joints (DAS28)), C-reactive protein levels (as a measure of systemic inflammation), Health Assessment Questionnaire scores (as a measure of disability), disease duration (early vs established; using 1 year as the differentiator as this is a commonly used cut-off and was also the median disease duration in our sample), sex and age were examined for their potential impact on bone density changes in response to GC treatment.

### Ancillary analyses

An additional post hoc analysis used data from one trial that could provide information on the use of anti-osteoporotic drugs (Glucocorticoid Low-dose Outcome in Rheumatoid Arthritis, GLORIA trial^7^). Here, participants receiving GCs who used an anti-osteoporotic drug at baseline were compared with participants receiving GCs who did not use an anti-osteoporotic drug at baseline.

### Sensitivity analyses

Sensitivity analyses, mostly concerning the handling of missing data, were conducted to assess the robustness of our results:

1. Repeating the main analysis with as-observed instead of imputed data (‘complete case analysis’).
2. Conducting the main analysis with 100 instead of five imputations.
3. Performing the main analysis with a linear mixed model (instead of ANCOVA), with time and a time×treatment interaction as covariates, using as-observed data.

### Multiplicity

The number of statistical tests was kept at a minimum: Tests for statistical significance were only conducted for the three coprimary endpoints. Bonferroni-corrected p values were provided for the main analyses of our three coprimary endpoints in line with a comment made in peer review.

### Patient and public involvement

Patients and members of the public were not involved in this study.

## Results

**Figure 1:**
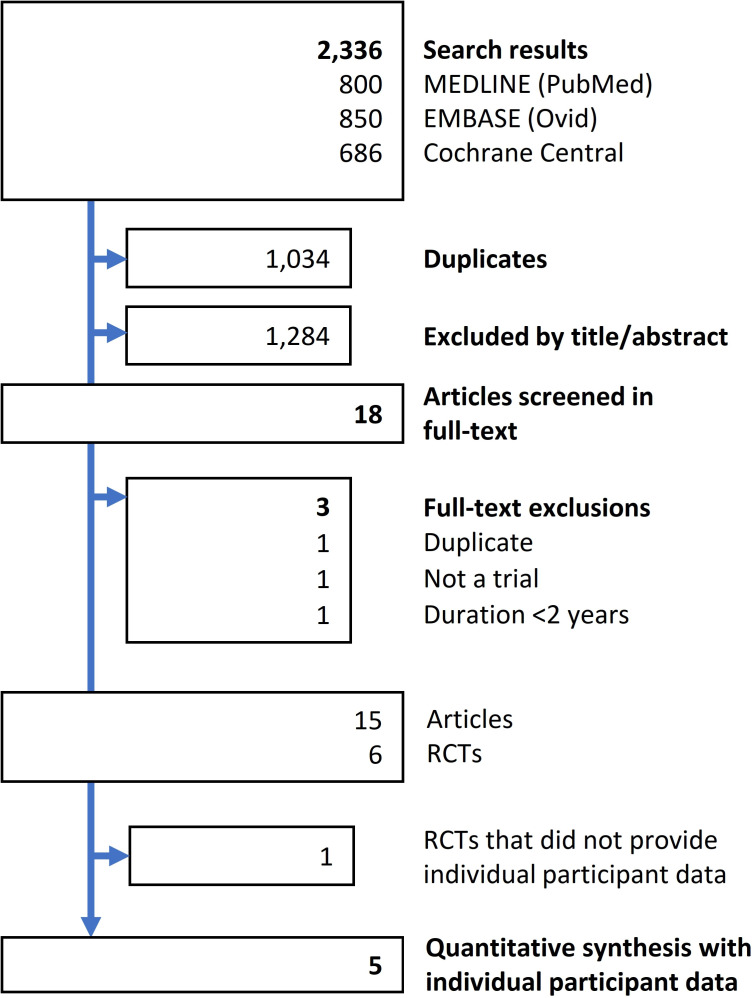
Search flow chart. RCTs, randomised controlled trials.

Our search yielded 2336 hits (figure 1). Based on 15 potentially eligible articles, corresponding to six trials that were scrutinised, five RCTs were ultimately included in the quantitative evidence synthesis:

1. GLORIA trial.^7^
2. Intramuscular Methylprednisolone Trial.^18^
3. Low-Dose Prednisolone Therapy Trial.^19^
4. Better Anti-Rheumatic Pharmacotherapy Trial (BARFOT).^20^
5. The Arthritis and Rheumatism Council Low-Dose Glucocorticoid Trial.^21^

All studies had a duration of 2 years, and all allowed concomitant treatment with DMARDs. Two studies investigated the effect of low-dose (7.5 mg/day prednisone equivalent)^20 21^ and three of very low-dose GCs (5 mg/day; one used 120 mg monthly depot intramuscular methylprednisolone injections.^7 18 18 19^ In the BARFOT trial,^20^ nine patients who did not receive any study medication could not be included in the intention-to-treat analyses as no data at all was available. One trial^22^ met the eligibility criteria (7 mg/day prednisolone vs placebo; 2-year duration), but IPD could not be obtained as the primary investigator did not respond to our repeated enquiries. In the published manuscript of this trial, bone density values had unclear units and were not presented with CIs or standard errors, so it was not possible to use these data as part of a two-stage analysis. Flow charts of all trials can be found in online supplemental figures S1–S5. IPD corresponded to the published trial reports if relevant numbers were reported, but this was not the case for all trials (eg, sometimes fracture numbers were not reported). ‘As observed’ endpoint data are shown separately for each endpoint and trial in the online supplemental table S3.

### Baseline characteristics

1112 participants (68% female) with a mean age of 61.4 years (±14.5) were included. The GLORIA trial^7^ enrolled most patients (n(randomised)=451), followed by the BARFOT^20^ trial (n(randomised)=250). Baseline characteristics, including lumbar spine and femoral bone density, were well balanced across groups (table 1). Baseline characteristics, stratified by trial, can be found in online supplemental table S4. Missingness for baseline and follow-up is shown in the online supplemental tables S3 and S5: Approximately half of the bone density data and a quarter of the fracture data were missing. While four trials provided femoral neck bone density data, one trial^7^ instead had total hip data available. Most patients were seropositive and had never or formerly smoked. The average participant was overweight and had elevated serological inflammatory markers. Mean DAS28 and Health Assessment Questionnaire scores categorised patients to have moderate disease activity and moderate to severe disability at baseline. Data on anti-osteoporotic medication was only available for the GLORIA trial^7^: At baseline, 13% were treated with specific anti-osteoporotic drugs (bisphosphonates or denosumab).

**Table 1:** Baseline characteristics, combined across trials

	Glucocorticoids n=548	Control n=564
Age, years	60.7 (14.8)	62.0 (14.2)
Female	346 (69.2)	345 (66.4)
ACPA positive	193 (62.3)	209 (65.7)
RF positive	324 (65.9)	343 (67.4)
Smoking status		
Never smoked	155 (45.2)	158 (44.5)
Previous smoker	119 (34.7)	128 (36.1)
Current smoker	69 (20.1)	69 (19.4)
BMI, kg/m^2^	26.4 (4.5)	26.6 (4.4)
Weight, kg	73.0 (13.5)	73.6 (15.2)
DAS28, score	4.8 (1.2)	5.0 (1.2)
Disease duration, years	1.0 (0.4–8.0) 5.9 (9.0)	1.0 (0.5–7.0) 5.5 (8.7)
Pain (0–10)	5.2 (2.4)	5.2 (2.3)
ESR, mm/h	30 (16–48) 35.2 (25.0)	30 (16–48) 34.9 (23.9)
CRP, mg/L	10.0 (3.5–23.5) 19.8 (27.1)	10.0 (4.0–28.0) 23.7 (32.1)
HAQ, score	1.5 (0.8)	1.4 (0.9)
BMD lumbar spine, g/cm²	1.08 (0.20)	1.07 (0.20)
BMD femur, g/cm²	0.88 (0.15)	0.88 (0.16)

Values are mean (SD), median (IQR) or n (%). Percentages are based on patients with available data. Values are based on individual participant data that were available to the study team. Pain was measured on the Visual or Numerical Analogue Scale. In one trial, a positive latex agglutination test was considered positive for rheumatoid factor.^21^ In one trial, the Hannover Functional Ability Questionnaire (FFbH) was used to evaluate disability—the scores were converted to HAQ scores according to the original publication with the following formula: HAQ=3.16–(0.028×FFbH).^19^

ACPA, anti-citrullinated protein antibody; BMD, bone mineral density; BMI, body mass index; CRP, C-reactive protein; DAS28, Disease Activity Score 28 joints; ESR, erythrocyte sedimentation rate; HAQ, Health Assessment Questionnaire; RF, rheumatoid factor.

### Bone mineral density

Both groups experienced bone loss at the lumbar spine and at the femur over 2 years (table 2). However, bone density decreased significantly more with GCs than with control treatment at the lumbar spine (difference between groups −0.021 g/cm²; 95% CI −0.037 to −0.005; p=0.034) but not at the femur (difference 0.004 g/cm²; 95% CI −0.008 to 0.016; p=0.47). There was little heterogeneity across trials and I² values were <32% for all endpoints. After Bonferroni correction, the difference in lumbar spine BMD did not meet the threshold for statistical significance anymore (online supplemental table S6; p=0.10).

**Table 2:** 2-year changes in bone density at the lumbar spine and the femur and the number of fractures in the glucocorticoid and control groups*

	Glucocorticoids (n=548)	Control (n=564)	Difference (95% CI)	P value	I²†
Change in lumbar spine BMD, g/cm²	−0.031 (0.009)	−0.010 (0.009)	−0.021 (−0.037 to −0.005)	0.034	31%
Change in femur BMD, g/cm²	−0.014 (0.006)	−0.018 (0.006)	0.004 (−0.008 to 0.016)	0.47	0%
Participants with fractures during the study period, no. (%)	26/548 (4.7%)‡	30/564 (5.3%)‡	0.86 (0.44 to 1.71)§	0.68	0%

* Missing data were handled using multiple imputation. Values are reported as least squares means (SE) unless otherwise stated in the table.

† I² signifies the percentage of variation across studies due to heterogeneity between trials rather than due to chance. It stems from additionally computed two-stage models.

‡ Values are n/N (%). Nota bene: These are not the numbers actually observed but the numbers estimated from multiple imputation.

§ Values are OR (95% CI).

BMD, bone mineral density.

### Fractures

Across trials, a total of 35 participants experienced at least one clinical fracture over 2 years (17 in the GC group and 18 in the control group were observed; 26 and 30, respectively, were estimated for the imputed dataset). Risks were similar in both groups: OR 0.86; 95% CI 0.44 to 1.71; p=0.68.

### Subgroup analyses

**Figure 2:**
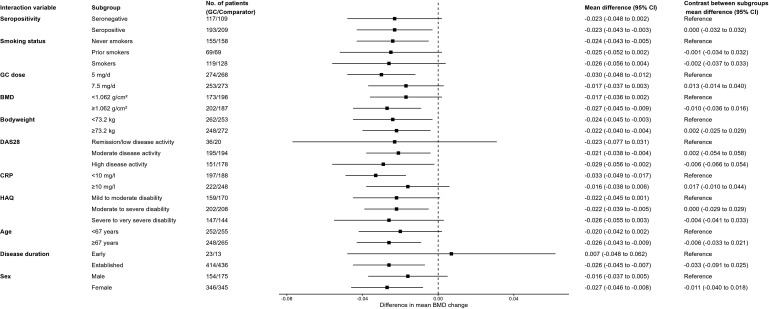
Subgroup analyses of changes in BMD of the lumbar spine. BMD, bone mineral density; CRP, C-reactive protein; DAS28, Disease Activity Score 28 joints; GC, glucocorticoid; HAQ, Health Assessment Questionnaire.

Results of the subgroup analyses investigating BMD of the lumbar spine using data from all trials according to various baseline characteristics are shown in figure 2. None of the variables investigated had a substantial effect on the impact of GCs on lumbar spine bone density, and CIs of different subgroups overlapped to a large extent.

### Ancillary analyses

In the GLORIA trial,^7^ anti-osteoporotic drug use was documented in a detailed manner. Of the 226 participants randomised to GC, 28 used an anti-osteoporotic drug at baseline (all either bisphosphonates or denosumab). BMD at the lumbar spine increased over 2 years in these patients (estimate: 0.01 g/cm²; 95% CI −0.03 to 0.06), while it decreased in the participants who were randomised to GC and did not use anti-osteoporotic drugs at baseline (n=198; estimate: −0.01 g/cm²; 95% CI −0.03 to 0.01).

### Sensitivity analyses

All sensitivity analyses (complete case analysis, linear mixed model, ANCOVA with 100 imputations) yielded results similar to the original main analysis (online supplemental tables S7–9).

## Discussion

In this systematic literature review and meta-analysis of IPD, we found low-dose GCs, taken over 2 years for the treatment of RA, to decrease bone density at the lumbar spine but not at the femur. Only a few fractures were observed, and the difference between GC and control groups was not statistically significant.

GCs have been playing a fundamental role in the treatment of RA for decades. They are still commonly used around the world (eg, in the USA,^1^ Canada,^23^ Australia,^24^ France,^25^ Germany,^26^ China^27^). However, current recommendations diverge greatly. The American College of Rheumatology conditionally recommends against using GCs when initiating a DMARD,^3^ but the EULAR recommends considering short-term GCs.^4^ A EULAR task force came to the conclusion that there is an acceptably low level of harm in most patients requiring a low GC dose (ideally, ≤ 5 mg/day prednisone equivalent).^5^ These differing recommendations are due to inconclusive evidence regarding GC-related adverse events. Observational studies mostly show an increase in various adverse events (eg, cardiovascular events, infections) even with low-dose GCs, but these results must be interpreted cautiously as patients with more severe disease are more likely to suffer from adverse events and to be prescribed GCs. We found that most observational studies investigating GC-related adverse events did not adequately adjust for confounders.^12^ This was even true for the subset of studies published in journals with a 2022 Journal Impact Factor ≥10. Additionally, even with adjustment for confounders such as disease activity, there may be residual confounding, which is why researchers investigating breast cancer treatments called confounding by indication a ‘most stubborn bias’.^28^ In a recent study, we revealed that observational studies had repeatedly overestimated the amount of blood pressure increase and weight gain with low-dose GCs^13^ in patients with RA.

Osteoporosis was among the most worrisome GC-related adverse events in a survey of rheumatologists and patients.^6^ Whether and to what extent low-dose GCs increase fracture risk and induce bone loss is controversially debated in the literature.^11^ Two cohort studies using a large UK primary care database associated low-dose GCs in RA with vertebral^10^ and other^29^ fractures but did not take into account confounders such as disease activity. In a large monocentric cross-sectional study investigating patients with RA from Germany (n=434), GCs were associated with impaired bone density, but only at moderate to high doses and only in conjunction with moderate or high disease activity.^8^ Similarly, in a US claims database analysis of patients with new-onset RA, only doses of ≥15 mg/day prednisone equivalent were associated with osteoporosis-related fractures.^9^

In our study, even low-dose GCs had a small but statistically significant effect on lumbar spine, but not on femoral bone density. Losing an additional 0.02 g/cm² (on average) due to GC over 2 years with a baseline value of 1.08 g/cm² in the GC group translates into an additional bone loss of 1.9% at the lumbar spine over 2 years. Assuming a linear trend, this would mean an additional bone loss of roughly 1% per year at the lumbar spine due to low-dose GC use. In comparison, in a multiethnic cohort of women, the average postmenopausal bone loss per year at the lumbar spine was 0.02 g/cm² or 2%.^30^ In another study, the average yearly decline in vertebral bone density was 0.01 g/cm² or 1% per year in the perimenopausal period (47 through 63 years), followed by a much slower decline of 0.005 g/cm² (0.5%) afterwards.^31^ From our point of view, the clinical relevance of an annualised 1% lumbar spine bone loss with low-dose GCs depends heavily on patient-specific (risk) factors. For example, in a young man with normal BMD and no other risk factors for osteoporosis other than RA, a 1% lumbar spine bone loss over 1 year could be much more acceptable than in a postmenopausal woman with a history of osteoporosis and prior vertebral fractures. Additionally, it remains unclear whether bone loss with low-dose GCs continues at a similar rate during longer-term treatment, so clinicians must be wary of patients receiving GCs for more than 2 years.

Regarding randomised evidence on low-dose GCs, prior RCTs did, in part, not report bone-related data in the main published manuscript.^21^ The largest and most recent trial^7^ reported results similar to our meta-analysis: a small but statistically significant decrease in lumbar spine bone density with low-dose GCs, no difference in total hip bone density. While RCTs offer protection from confounding, they are usually powered to show a contrast in the primary outcome measure (mostly pertaining to efficacy), and participant numbers are mostly smaller than in observational research. A solution to this conundrum is to perform a meta-analysis of RCTs, where methodological advantages of trials remain while statistical power increases due to larger overall sample sizes. Using IPD offers additional advantages^32^: the extent of missing data can be estimated and its effect may be addressed in statistical analyses; data that have not been published in sufficient detail can be included; statistical analysis methods can be standardised for the different included studies; prerequisites for statistical models can be evaluated; adjustment for baseline factors is possible if randomisation was not completely successful; and subgroup analyses can be conducted (eg, for patients with risk factors).

In our study, we planned to identify subgroups especially vulnerable to GC-induced bone loss in order to guide rheumatologists as to which patients might need closer monitoring or earlier treatment with anti-osteoporotic drugs. However, subgroup analyses did not reveal substantial differences between groups, and very small differences with overlapping CIs might be due to chance. To our knowledge, there is only one other relevant study: we performed a cross-sectional study of 483 patients with RA, but neither age nor sex modified the association between GCs and bone density.^33^ In summary, low-dose GCs do not seem to affect certain subgroups of RA patients more than others. Therefore, we recommend that clinicians should consider classical risk factors of osteoporosis, bone loss and fractures such as low body weight and advanced age when considering fracture risk, screening and treatment in all patients with RA.^11^

Interestingly, in the GLORIA trial, the overall trial population had a slightly positive change in BMD, both at the lumbar spine and the femur (see online supplemental table S3). This was in contrast to other trials included in our study. We can only speculate on reasons, but this effect might be the result of one of several differences in GLORIA compared with the other trials: The GLORIA trial is the most contemporary of all included trials; it was very lenient regarding cotreatment with other drugs, and biologic DMARDs were available in this trial. As a consequence, patients may have had better disease control compared with other trials, which could have been beneficial to BMD.^11^ Additionally, because of the growing recognition of osteoporosis as a significant comorbidity, clinicians might have paid more attention to optimising relevant risk factors. For example, 81% received calcium and vitamin D supplementation.

Finally, in a post hoc analysis, we assessed the effect of anti-osteoporotic medications on GC-induced bone loss at the lumbar spine in a subgroup of the GLORIA trial. In this trial, comedication was meticulously documented. While lumbar spine bone density decreased with GCs over 2 years in patients without anti-osteoporotic medication at baseline, there was even a slight increase in patients taking either bisphosphonates or denosumab at baseline (teriparatide and romosozumab were not used at all), suggesting these drugs protect from GC-induced bone loss. Our analysis conforms with prior studies of, for example, alendronate^34^ or denosumab^35^ in GC-induced osteoporosis.

This study has a number of limitations. First, fractures are the clinical outcome of interest, but only very few occurred in our study, probably leading to insufficient statistical power. However, we were able to gather data on BMD for a large number of patients, and changes in bone density are known to correlate well with fracture risk.^36^ Second, some of the included trials were older, conducted before modern therapies such as biologic or targeted synthetic DMARDs became available. This might have led to worse disease control and possibly worse bone health in the control groups. The majority of patients in our meta-analysis, though, stemmed from the GLORIA trial, which was published in 2022 and included patients using modern treatments like biologic DMARDs. Third, the information on anti-osteoporotic treatments was sparse. Possibly, patients in the GC group who experienced bone loss were prescribed anti-osteoporotic drugs during the study, which in turn might have attenuated the effect of low-dose GCs on bone density, meaning the effect of GCs on bone density might be stronger than observed in this study. Fourth, all trials were conducted in European countries, thereby limiting the generalisability to other populations. Fifth, some of the included trials were conducted before guidelines on preventing GC-induced osteoporosis took effect. Sixth, bone density measurements were performed with different devices, leading to more ‘noise’ in the data. However, we accounted for differences across trials by including trial ID in our statistical models. Seventh, as there was no standardised screening method for vertebral fractures (which may cause very little to no symptoms), it is probable that we have missed some. In the GLORIA trial,^7^ there was a statistically non-significant increase in image-detected spine compression fractures with low-dose GCs, which corresponds to the decrease in lumbar spine bone density observed in our study. Eighth, no data on vitamin D supplementation or serum vitamin D levels were available. Small trials found that vitamin D supplementation might mitigate negative bone-related GC effects.^37 38^ If patients in the GC groups were more likely to use vitamin D supplements, the true effect of GC on bone density and fractures might be stronger than seen in our analyses. Ninth, we were not able to acquire data from one trial^22^ which met the eligibility criteria because the primary investigator did not respond to our enquiries, and aggregate data could not be integrated due to the reasons outlined in the results section. According to the published data of this trial, over 2 years, patients in the GC group had a more pronounced bone loss at the lumbar spine and more patients in the GC group initiated bisphosphonates. Finally, a substantial proportion of data was missing, which could have impacted our findings, but several relevant sensitivity analyses, including an analysis of the data as observed, yielded similar results as the main multiply imputed analysis.

## Conclusions

In conclusion, in patients with RA, 2 years of treatment with low-dose GCs leads to bone loss at the lumbar spine but not at the femur. From the authors’ viewpoint, the clinical relevance of the bone loss observed at the lumbar spine depends on patient-specific risk factors for osteoporosis.

## Supplementary material

10.1136/rmdopen-2025-006615 online supplemental appendix 1

## Data Availability

No data are available.
