# Supplementary material for: Changes in bone mineral density and fractures during 2 years of low-dose glucocorticoid treatment for rheumatoid arthritis: a systematic literature review and individual participant data meta-analysis
Source: RMD Open. 2026 May 7;12(2):e006615. doi: 10.1136/rmdopen-2025-006615 (PMC13157747; doi:10.1136/rmdopen-2025-006615)
Supplement: online supplemental appendix 1 [file rmdopen-12-2-s001.docx]

**Appendix to**

**Changes in Bone Mineral Density and Fractures During Two Years of Low Dose Glucocorticoid Treatment for Rheumatoid Arthritis:
A Systematic Literature Review and Individual Participant Data Meta-Analysis**

Andriko Palmowski1,2,9,10, Tobias Haugegaard², Ingiäld Hafström3, Henning Bliddal2, Judith Oldenkott1, Siegfried Wassenberg4, Ernest Choy5, John Kirwan6, Robin Christensen2,7, Maarten Boers8, Frank Buttgereit1,11

1 Department of Rheumatology and Clinical Immunology, Charité – University Medicine Berlin, Germany
2 Section for Biostatistics and Evidence-Based Research, the Parker Institute, Bispebjerg and Frederiksberg Hospital, Frederiksberg, Denmark
3 Division of Gastroenterology and Rheumatology, Department of Medicine Huddinge, Karolinska Institutet, Stockholm, Sweden
4 Rheumazentrum Ratingen, Ratingen, Germany
5 Cardiff University, UK
6 University of Bristol, UK
7 Research Unit of Rheumatology, Department of Clinical Research, University of Southern Denmark, Odense University Hospital, Odense, Denmark

8 Department of Epidemiology and Data Science, Amsterdam UMC, The Netherlands
^9^ Epidemiology and Health Services Research, German Rheumatism Research Center (DRFZ), an Institute of the Leibniz Association, Berlin, Germany

10 Berlin Institute of Health (BIH) at Charité – Universitätsmedizin Berlin, Berlin, Germany

11 Translational Rheumatology, German Rheumatism Research Center (DRFZ), A Leibniz Institute, Berlin, Germany

**Supplementary Table S1.** Important protocol deviations.

| **Deviation** | **Explanation** |
| --- | --- |
| References of included studies were screened. | This was done to identify more eligible articles. |
| Initially, a combined analysis for spinal and femoral bone density was planned in the protocol using the lowest value of either the femur or lumbar spine, but lumbar spine and femur bone density were analysed separately. | We had not expected many patients to have received bone density measurements of both the lumbar spine and the femur. As the numbers for both measurement sites were higher than expected, and because GCs are known to affect these sites differently, we decided – before any analysis was initiated – to perform separate analyses for lumbar spine and femoral bone density. |
| For subgroup analyses, a combined analysis using the lowest score of either lumbar spine or the femoral bone density measurement was planned. | We decided to assess lumbar spine bone density only, as the lumbar spine is known to be affected by GCs to a greater extent, and as more measurement results were available than originally expected. |
| Subgroup analyses included disease duration (early vs. established RA) and sex. | Ideas for both subgroups came up during peer review. |
| Baseline characteristics are shown stratified by trial in a supplementary table. | Suggestion during peer review. |
| Means ± SD are reported for variables not following a normal distribution. | Suggestion during peer review. |
| Various additional sensitivity analyses | Suggestion during peer review. |

**Supplementary Table S2.** Search strings for MEDLINE, EMBASE, and Cochrane CENTRAL.

| **MEDLINE (via PubMed)**  (Rheumatoid [Title] OR Arthritis, Rheumatoid [MESH:NoExp])  AND  (Alclometason*[TiAb] or Amcinonid*[TiAb] or Beclomethas*[TiAb] or Beclomethason*[TiAb] or  Betamethason*[TiAb] or Budesonid*[TiAb] or Clobetaso*[TiAb] or Clocortolon*[TiAb] or  Clopredno*[TiAb] or Cortison*[TiAb] or Cortivazo*[TiAb] or Deflazacor*[TiAb] or  Desoximetason*[TiAb] or Dexamethason*[TiAb] or Dichlorison*[TiAb] or Diflorason*[TiAb] or  Diflucortolon*[TiAb] or Difluprednat*[TiAb] or Flumethason*[TiAb] or Fluocinolon*[TiAb] or  Fluocinonid*[TiAb] or Fluocorti*[TiAb] or Fluocortolon*[TiAb] or Fluorometholon*[TiAb] or  Flupredniden*[TiAb] or Fluprednisolon*[TiAb] or Flurandrenolon*[TiAb] or Fluticason*[TiAb] or  Hydrocortison*[TiAb] or Medryson*[TiAb] or Melengestro*[TiAb] or Meprednison*[TiAb] or  Methylprednisolon*[TiAb] or Paramethason*[TiAb] or Prednicarbat*[TiAb] or Predniso*[TiAb] or  Rimexolon*[TiAb] or Triamcinolon*[TiAb] or Cortico*[TiAb] or Glucocort*[TiAb] or Steroid*[TiAb] or Glucocorticoids[MeSh:NoExp])  AND  (Trial[Title] OR Randomi*[Title] OR Controlled[Title] OR Randomized Controlled Trial[Publication Type:NoExp])  **EMBASE (via Ovid)**  (Rheumatoid.ti or rheumatoid arthritis/)  AND  ((Alclometason* or Amcinonid* or Beclomethas* or Beclomethason* or Betamethason* or Budesonid* or Clobetaso* or Clocortolon* or Clopredno* or Cortison* or Cortivazo* or Deflazacor* or Desoximetason* or Dexamethason* or Dichlorison* or Diflorason* or Diflucortolon* or Difluprednat* or Flumethason* or Fluocinolon* or Fluocinonid* or Fluocorti* or Fluocortolon* or Fluorometholon* or Flupredniden* or Fluprednisolon* or Flurandrenolon* or Fluticason* or Hydrocortison* or Medryson* or Melengestro* or Meprednison* or Methylprednisolon* or Paramethason* or Prednicarbat* or Predniso* or Rimexolon* or Triamcinolon* or Cortico* or Glucocort* or Steroid*).ti,ab OR glucocorticoid/)  AND  ((Trial OR Randomi* OR Controlled).ti)  **Cochrane** **CENTRAL**  #1 MeSH descriptor: [Arthritis, Rheumatoid] this term only  #2 rheumatoid:ti  #3 #1 OR #2  #4 (Alclometason* or Amcinonid* or Beclomethas* or Beclomethason* or Betamethason* or  Budesonid* or Clobetaso* or Clocortolon* or Clopredno* or Cortison* or Cortivazo* or  Deflazacor* or Desoximetason* or Dexamethason* or Dichlorison* or Diflorason* or Diflucortolon* or Difluprednat* or Flumethason* or Fluocinolon* or Fluocinonid* or Fluocorti* or Fluocortolon* or Fluorometholon* or Flupredniden* or Fluprednisolon* or Flurandrenolon* or Fluticason* or Hydrocortison* or Medryson* or Melengestro* or Meprednison* or Methylprednisolon* or Paramethason* or Prednicarbat* or Predniso* or Rimexolon* or Triamcinolon* or Cortico* or Glucocort* or Steroid*):ti,ab  #5 MeSH descriptor: [Glucocorticoids] this term only  #6 #4 OR #5  #7 (Trial OR Randomi* OR Controlled):ti  #8 ("randomized controlled trial"):pt  #9 #7 or #8  #10 #3 AND #6 AND #9 |
| --- |


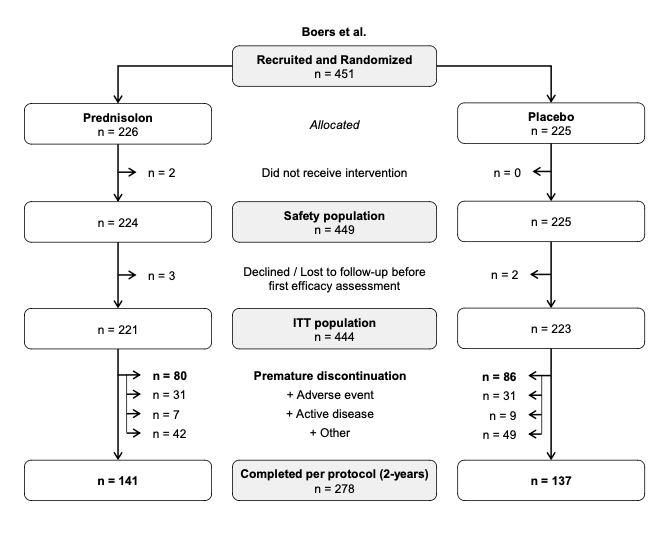
**Flow Charts**


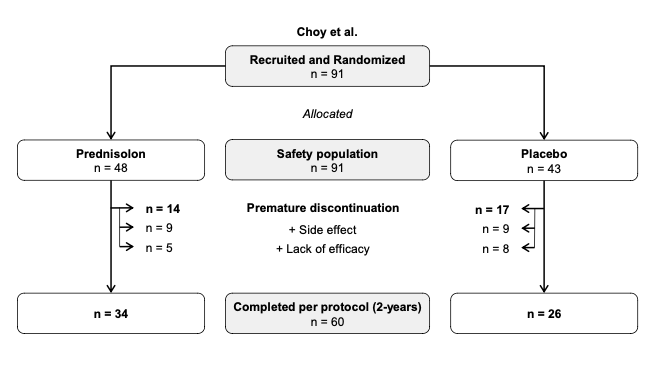
**Supplement Figure S1.** Flow chart of the Boers et al. trial.

**Supplement Figure S2.** Flow chart of the Choy et al. trial.


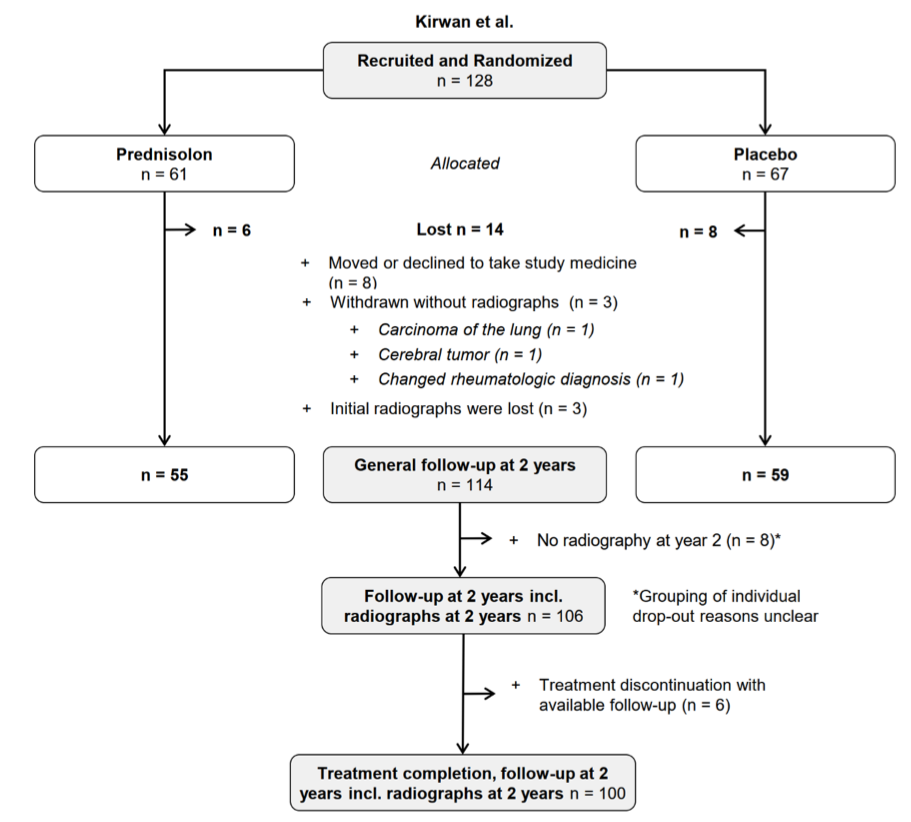
**Supplement Figure S3.** Flow Chart of the Kirwan et al. trial.


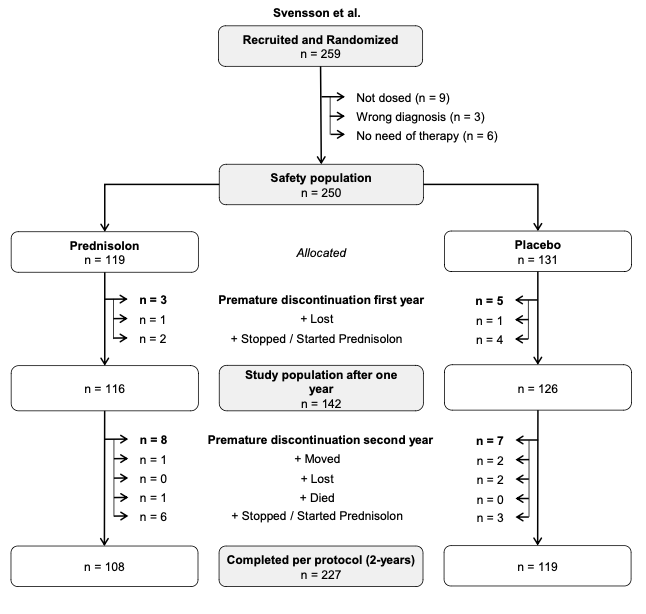
**Supplement Figure S4.** Flow Chart of the Svensson et al. trial.

**
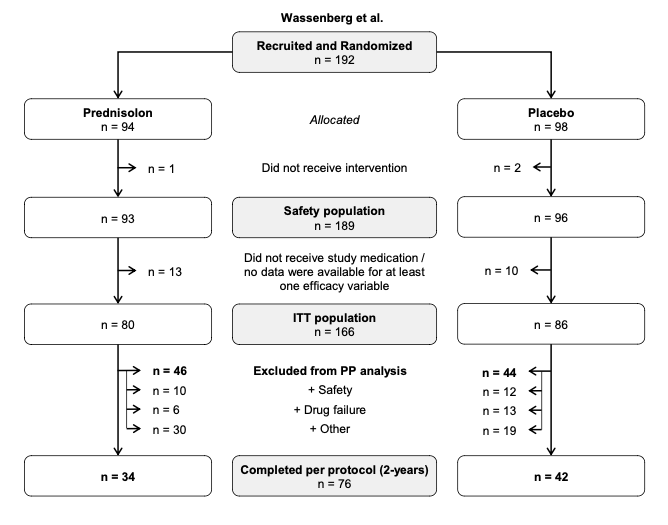
Supplement Figure S5.** Flow Chart of the Wassenberg et al. trial.

**Supplementary Table S3.** Endpoint data (as observed), presented separately for each trial.

| Endpoint | Trial ID | n | % observed (not missing) | Result* |
| --- | --- | --- | --- | --- |
| Change in lumbar spine BMD, g/cm² | Boers | 266 | 59.0 % | 0.006 ± 0.08 |
|  | Choy | 15 | 16.5 % | -0.026 ± 0.06 |
|  | Wassenberg | 18 | 9.4 % | -0.049 ± 0.05 |
|  | Svensson | 168 | 67.2 % | -0.018 ± 0.06 |
|  | Kirwan | 42 | 32.8 % | -0.048 ± 0.06 |
| Change in femur BMD, g/cm² | Boers | 247 | 54.8 % | 0.002 ± 0.09 |
|  | Choy | 13 | 14.3 % | -0.056 ± 0.05 |
|  | Wassenberg | NA | NA | NA |
|  | Svensson | 198 | 79.2 % | -0.019 ± 0.06 |
|  | Kirwan | 44 | 34.4 % | -0.035 ± 0.06 |
| Participants with fractures during the study period, no. (%) | Boers | 285 | 63.2 % | 23 (8.1 %) |
|  | Choy | 57 | 62.6 % | 0 (0.0 %) |
|  | Wassenberg | 106 | 55.2 % | 5 (4.7 %) |
|  | Svensson | 248 | 99.2 % | 6 (2.4 %) |
|  | Kirwan | 112 | 87.5 % | 1 (0.9 %) |
| BMD, bone mineral density; ID, identifier; NA, not available. *Numbers are based on as observed data and signify mean ± standard deviation or n (%). | | | | |

| Supplementary Table S4. Baseline patient characteristics stratified by trial. | | | | | |
| --- | --- | --- | --- | --- | --- |
| First author  Year  (Sample size) | **Boers**  **2022**  (451) | **Choy  2005**  (91) | **Kirwan  1995**  (128) | **Svensson  2005**  (250) | **Wassenberg  2005**  (192) |
| Age, years | 72 (68; 76) | 58 (± 12)* | 48 (42; 58) | 56 (46; 66) | 56 (41; 62) |
| Female | 317 (70 %) | 71 (78 %)* | 81 (63 %) | 159 (64 %) | 134 (70 %) |
| Seropositivity |  |  |  |  |  |
| ACPA positive | 253 (64 %) | NA | NA | 149 (64 %) | NA |
| RF positive | 299 (68 %) | 47 (59%)*; † | 102 (86 %)‡ | 163 (66 %) | 103 (54 %) |
| Smoking status |  |  |  |  |  |
| Never | 221 (49 %) | NA | NA | 92 (37 %) | NA |
| Current | 62 (14 %) | NA | NA | 76 (31 %) | NA |
| Previous | 166 (37%) | NA | NA | 81 (33 %) | NA |
| BMI, kg/m^2^ | 27.3 (± 4.5) | NA | NA | 25.8 (± 4.2) | 25.7 (± 4.5) |
| DAS28 | 4.52 (± 1.05) | 5.39 (± 1.29) | NA | 5.35 (± 1.08) | NA |
| Disease duration, years | 7.0 (3; 15) | 14 (± 9)* | 1.3 (± 0.29)* | 0.5 (0.3; 0.7) | 0.5 (0.3; 0.1) |
| Pain, score | 5.5 (± 2.4) | 4.6 (± 2.4) | 4.8 (± 2.5) | 4.8 (± 2.2) | 5.7 (± 2.2) |
| ESR, mm/h | 25.0 (14; 40) | 26.0 (11; 40.5) | 45.0 (26; 60) | 32.5 (18; 52) | 38.0 (24; 60) |
| CRP, mg/l | 5.0 (2.7; 13.7) | 11.1 (5; 28.2) | 19.0 (10; 36) | 23.0 (10; 52) | NA |
| HAQ | 1.25 (0.63; 1.75) | 1.75 (1; 2)§ | 1.3 (0.7; 1.8) | 1.0 (0.5; 1.4) | NA\|\| |
| Weight, kg | 74.8 (± 13.5) | 71.0 (± 21.7) | 69.2 (± 13.7) | 74.0 (± 13.9) | 72.2 (± 14.2) |
| Numbers are n (%), mean (standard deviation), or median (interquartile range), all based on individual participant data that were available to the study team. *Numbers not available on an individual level (extracted from published aggregate data). †Rheumatoid factor status known in 80 individuals. ‡Positive latex agglutination test. §Modified HAQ was used in this study. \|\|The Functional Questionnaire Hanover (FFbH) was used in this study to evaluate disability. ACPA = antibody citrullinated protein antibodies; BMI = body mass index; CRP = C-reactive protein; DAS28 = Disease Activity Score 28 joints; ESR = erythrocyte sedimentation rate; HAQ = Health Assessment Questionnaire; NA = not available; RF = rheumatoid arthritis. | | | | | |

|  |  | % Missing |
| --- | --- | --- |
| Baseline | Age, years | 8 |
|  | Female | 8 |
|  | ACPA positive | 44 |
|  | RF positive | 10 |
|  | Smoking status | 37 |
|  | BMI, kg/m^2^ | 21 |
|  | Weight, kg | 4 |
|  | DAS28, score | 30 |
|  | Disease duration, years | 20 |
|  | Pain (0-10) | 4 |
|  | ESR, mm/h | 7 |
|  | CRP, mg/l | 23 |
|  | HAQ, score | 5 |
|  | BMD lumbar spine, g/cm² | 30 |
|  | BMD femur, g/cm² | 33 |
| Follow-up (two years) | BMD lumbar spine, g/cm² | 53 |
|  | BMD femur, g/cm² | 54 |
|  | Fractures | 27 |
| Values are based on individual participant data that were available to the study team. Pain was measured on the visual or numerical analogue scale. In one trial, a positive latex agglutination text was considered positive for rheumatoid factor [18]. In one trial, the Hannover Functional Ability Questionnaire (FFbH) was used to evaluate disability – the scores were converted to HAQ scores according to the original publication with the following formula: HAQ = 3.16 – (0.028 x FFbH) [16]. ACPA, anti-citrullinated protein antibody, RF, rheumatoid factor, BMI, body mass index, DAS28, disease activity score 28 joints, ESR, erythrocyte sedimentation rate, CRP, C-reactive protein, HAQ, health assessment questionnaire, BMD, bone mineral density. | | |

**Supplementary Table S5.** Missing variables at baseline and follow-up.

| Supplementary Table S6. Bonferroni-corrected p-values for the original main analysis. | | | | | | |  |
| --- | --- | --- | --- | --- | --- | --- | --- |
|  |  |  |  |  |  | ***p*** | |
| **Change in lumbar spine BMD, g/cm²** |  |  |  |  |  | 0.10 | |
| **Change in femur BMD, g/cm²** |  |  |  |  |  | 1 | |
| **Participants with fractures during the study period, no. (%)** |  |  |  |  |  | 1 | |
| BMD = Bone Mineral Density. | | | | | | |  |

| Supplementary Table S7. Sensitivity analysis using data ‘as observed’ (‘complete case analysis’) instead of multiply imputed data. 2-Year changes in bone density at the lumbar spine and the femur and number of fractures in the glucocorticoid and control groups* | | | | | | |
| --- | --- | --- | --- | --- | --- | --- |
|  | **n** | **Glucocorticoids** | **n** | **Control group** | **Difference (95% CI)** | ***p*** |
| **Change in lumbar spine BMD, g/cm²** | 255 | -0.036 (0.011) | 254 | -0.011 (0.011) | -0.025 (-0.037 to -0.013) | <0.001 |
| **Change in femur BMD, g/cm²** | 255 | -0.018 (0.011) | 247 | -0.025 (0.011) | 0.006 (-0.006 to 0.019) | 0.33 |
| **Participants with fractures during the study period, no. (%)** | 396 | 17 (4.3%) † | 412 | 18 (4.4%) † | 0.96 (0.49 to 1.89) ‡ | 0.91 |
| 95%CI = 95% confidence interval; GC = Glucocorticoid; BMD = Bone Mineral Density.  * Values are reported as least squares means (standard error) unless otherwise stated.  † Values are N (%).  ‡ Values are OR (CI) | | | | | | |

| Supplementary Table S8. Sensitivity analysis using 100 instead of five imputations. 2-Year changes in bone density at the lumbar spine and the femur and number of fractures in the glucocorticoid and control groups* | | | | | | |
| --- | --- | --- | --- | --- | --- | --- |
|  |  | **Glucocorticoids** |  | **Control group** | **Difference (95% CI)** | ***p*** |
| **Change in lumbar spine BMD, g/cm²** |  | -0.029 (0.012) |  | -0.009 (0.012) | -0.021 (-0.033 to -0.009) | 0.005 |
| **Change in femur BMD, g/cm²** |  | -0.013 (0.009) |  | -0.022 (0.009) | 0.009 (-0.004 to 0.021) | 0.19 |
| **Participants with fractures during the study period, no. (%)** |  | 30 (5.5%) † |  | 29 (5.1%) † | 1.13 (0.58 to 2.19) ‡ | 0.73 |
| 95%CI = 95% confidence interval; GC = Glucocorticoid; BMD = Bone Mineral Density.  * Values are reported as least squares means (standard error) unless otherwise stated.  † Values are N (%).  ‡ Values are OR (CI) | | | | | | |

| Supplementary Table S9. Sensitivity analysis using a linear mixed model without imputation. 2-Year changes in bone density at the lumbar spine and the femur in the glucocorticoid and control groups* | | | | | | |
| --- | --- | --- | --- | --- | --- | --- |
|  |  | **Glucocorticoids** |  | **Control group** | **Difference (95% CI)** | ***p*** |
| **Change in lumbar spine BMD, g/cm²** |  | -0.026 (0.005) |  | -0.002 (0.005) | -0.024 (-0.032 to -0.016) | <0.001 |
| **Change in femur BMD, g/cm²** |  | -0.011 (0.004) |  | -0.017 (0.004) | 0.007 (-0.001 to 0.015) | 0.10 |
| 95%CI = 95% confidence interval; GC = Glucocorticoid; BMD = Bone Mineral Density.  * Values are reported as least squares means (standard error) unless otherwise stated. | | | | | | |
